# Pressure-induced structural transformations and polymerization in ThC_**2**_

**DOI:** 10.1038/srep45872

**Published:** 2017-04-06

**Authors:** Yongliang Guo, Cun Yu, Jun Lin, Changying Wang, Cuilan Ren, Baoxing Sun, Ping Huai, Ruobing Xie, Xuezhi Ke, Zhiyuan Zhu, Hongjie Xu

**Affiliations:** 1 Shanghai Institute of Applied Physics, Chinese Academy of Sciences, Shanghai 201800, China; 2 Institute of Theoretical Physics, Department of Physics, East China Normal University, Shanghai 200241, China; 3 Key Laboratory of Interfacial Physics and Technology, Chinese Academy of Sciences, Shanghai 201800, China

## Abstract

Thorium-carbon systems have been thought as promising nuclear fuel for Generation IV reactors which require high-burnup and safe nuclear fuel. Existing knowledge on thorium carbides under extreme condition remains insufficient and some is controversial due to limited studies. Here we systematically predict all stable structures of thorium dicarbide (ThC_2_) under the pressure ranging from ambient to 300 GPa by merging *ab initio* total energy calculations and unbiased structure searching method, which are in sequence of C2/c, C2/m, Cmmm, Immm and P6/mmm phases. Among these phases, the C2/m is successfully observed for the first time via *in situ* synchrotron XRD measurements, which exhibits an excellent structural correspondence to our theoretical predictions. The transition sequence and the critical pressures are predicted. The calculated results also reveal the polymerization behaviors of the carbon atoms and the corresponding characteristic C-C bonding under various pressures. Our work provides key information on the fundamental material behavior and insights into the underlying mechanisms that lay the foundation for further exploration and application of ThC_2_.

In order to achieve deep cuts in fossil fuel use while meeting the growing global demand for affordable, reliable energy, previous studies^1^,^2^,^3^,^4^ estimate that a doubling to quadrupling of nuclear energy output is required in the next few decades, along with a large expansion of renewable energy. Thorium, as the new frontier of nuclear energy, has attracted increasing interest worldwidely^5^. Several nations, including China, India and United States, are taking a close look at thorium as a nuclear fuel^5^,^6^. Thorium is three to four times more naturally abundant than uranium^6^,^7^,^8^. A thorium fuel cycle can be developed to produce negligible amounts of plutonium and fewer long-lived minor actinides compared to a uranium cycle^6^,^9^. Many attempts have been made to fabricate and measure thorium compounds as a potential fuel for advanced reactors.

Recently, the thorium-carbon system has attracted more and more attentions as these compounds are suitable for high-burnup and high-temperature operations with increased “margin to melting” in the frame-work of the Generation IV nuclear systems^10^. Thorium is known to form two main types of stoichiometric carbides: monocarbide (ThC) and dicarbide (ThC_2_). At ambient conditions, thorium monocarbide is of the ‘salt-like’ type structure, in which carbons present as single anions C^4−^. The evolution of the crystal structure under high pressure was investigated in our previous work^11^ as well as the work of Sahoo *et al*.^12^. Thorium dicarbide has three stoichiometric phases at ambient pressure^10^,^13^,^14^,^15^: monoclinic structure (space group C2/c), stable from room temperature to 1713 K; CaC_2_-tetragonal structure (space group I4/mmm), stable for 1713 K ≤ T ≤ 1768 K; KCN-fcc structure (space group 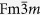), stable above 1768 K. In these phases, the carbons group in pairs as C-C dumbbells. The C-C bond in carbon dumbbell has strong covalent character and the carbons present as 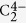 anions^13^,^16^.

Understanding the structural information is essential to explore the physical properties of materials as the latter normally depend very sensitively on the former^17^. It is therefore crucial to determine the correct structures of materials. Previous research suggests various crystalline structures of ThC under high pressure^11^,^12^,^18^. For ThC_2_, although its structures have been obtained at ambient pressure and high temperature^10^,^13^,^14^,^15^, the structural evolution under high pressure has not been fully understood until now. A clear understanding of the nuclear fuels’ stabilities under extreme conditions is very important for the safe operation of nuclear reactors. Here we report the possible structures of ThC_2_ under high pressure predicted by the first-principles total energy calculations and the particle-swarm optimization algorithm^19^. The structural details up to ~50 GPa are confirmed by the *in situ* synchrotron X-ray diffraction. The main purpose of this work is to explore the new crystal structures of the thorium dicarbide under high pressure and to clarify the mechanism of their phase transitions.

Our structure search theoretically predicts five viable crystal phases of ThC_2_ in C2/c, C2/m, Cmmm, Immm and P6/mmm symmetry, respectively, and the calculated phonon dispersions confirm their dynamical stability. The ground state phase C2/c and the predicted phase C2/m are successfully observed by *in situ* synchrotron XRD. Further studies of enthalpy and electronic density provide key information on the evolution of the structural and carbon-carbon bonding properties of these phases. The calculations also reveal the carbon-carbon polymerization characters and their effect on the vibrational behaviors. The present results establish key material properties of ThC_2_ that are important to both fundamental understanding and practical application of this interesting compound.

## Results

### Structures and phase transitions under pressure

**Figure 1:**
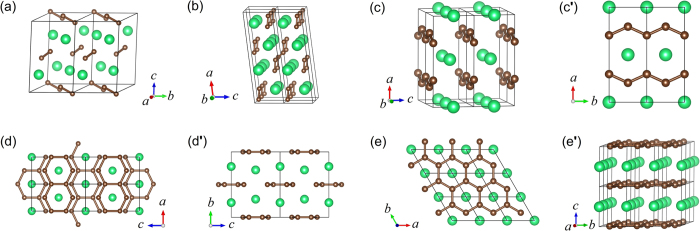
Theoretical predicted crystal structures of ThC_2_. (**a**) C2/c phase; (**b**) C2/m phase; (**c**,**c**′) Cmmm phase; (**d**,**d**′) Immm phase; and (**e**,**e**') P6/mmm phase. The green and brown balls represent Th and C atoms, respectively.

To explore all possible phases of ThC_2_ under high pressures, we perform an extensive structure search through the CALYPSO code^20^ without any presumed structural information at 0 GPa, 50 GPa, 100 GPa, 150 GPa and 200 GPa. Further analysis on enthalpy and stability lead to five possible structural states up to 300 GPa, as illustrated in Fig. 1, which are C2/c, C2/m, Cmmm, Immm and P6/mmm as pressure goes up.

Under ambient conditions, the ThC_2_ has a monoclinic structure (space group C2/c) with four formula units in unit cell^10^,^13^,^14^,^15^,^21^, as shown in Fig. 1(a). The crystal structure of ThC_2_ polymorph contains discrete C2 structural units — so-called C-C dumbbells, which exist commonly in alkaline-earth metal, transition metal, and lanthanide or actinide metal carbides. The experimentally measured C-C bond length in C2/c phase of ThC_2_ is 1.315 Å^14^ and 1.304 Å^15^, a typical double carbon bond as found in C_2_H_4_ (1.34 Å).

**Figure 2:**
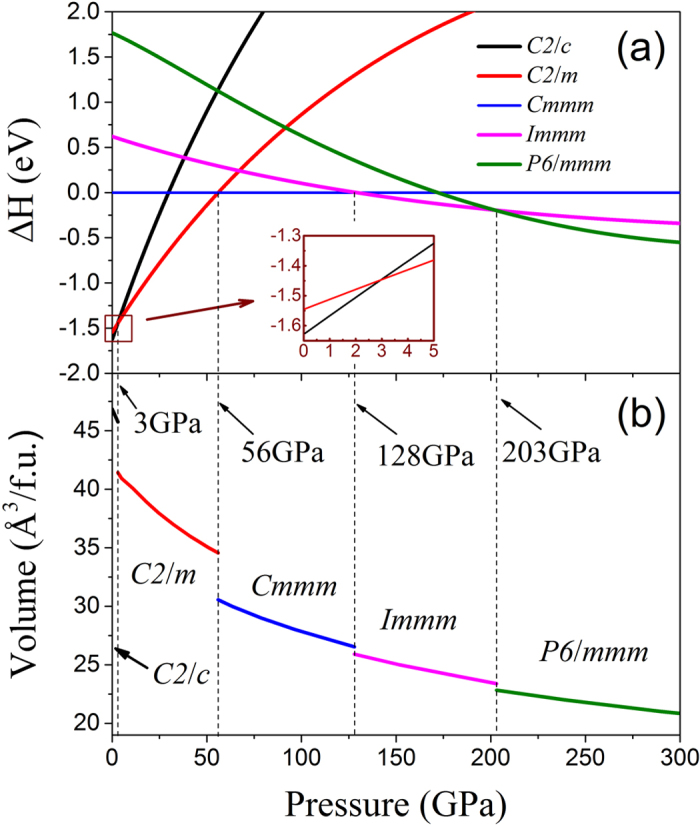
Calculated (**a**) enthalpies and (**b**) volumes per f.u. as a function of pressure for C2/c, C2/m, Cmmm, Immm and P6/mmm phases of ThC_2_. The enthalpy of Cmmm phase is chosen as zero reference. The inset of (**a**) is an enlarged detail view for the selected portion by rectangular box. The transition pressures are 3 GPa, 56 GPa, 128 GPa and 203 GPa, and the corresponding abrupt volume collapses are about 9.5%, 11.6%, 2.3% and 2.4%, respectively.

In order to obtain the transition pressure and transition sequence among these phases, the enthalpy differences as a function of pressure are calculated and presented in Fig. 2(a). It can be seen that the ambient-pressure C2/c phase is the most stable structure below 3 GPa; the C2/m phase becomes more favorable at the pressure ranging from 3 to 56 GPa; the Cmmm phase is stable from 56 to 128 GPa; the Immm phase is stable from 128 to 203 GPa, above which the P6/mmm phase is energetically favorite. The pressure-induced evolution of the unit cell volume for the various phases of ThC_2_ is calculated and shown in Fig. 2(b). The abrupt volume collapse through each transition are about 9.5%, 11.6%, 2.3% and 2.4% at around 3 GPa, 56 GPa, 128 GPa and 203 GPa, respectively, indicating that these transitions are of the first-order.

**Figure 3:**
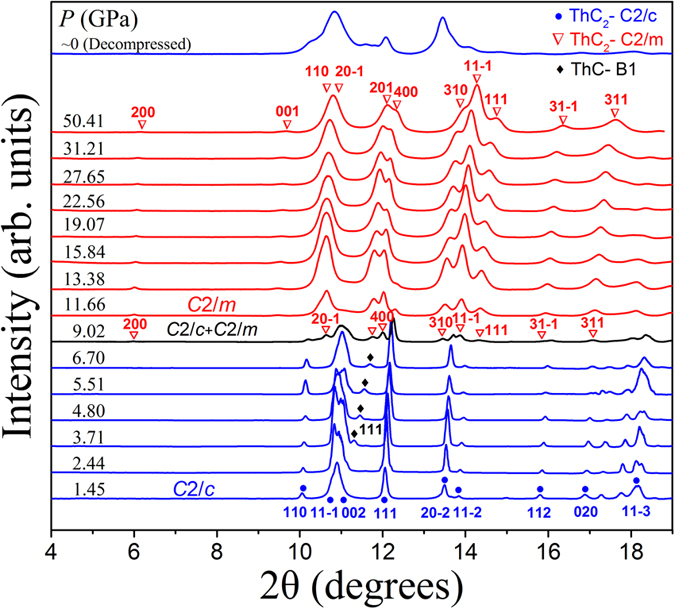
X-ray diffraction patterns of ThC_2_ as pressure increased and then decreased to ambient pressure (λ = 0.6199 Å, T = 300 K). The solid circles and hollow triangles mark the peaks associated with the C2/c and C2/m phases of ThC_2_, respectively. The diamonds mark the peaks associated with the B1 phase of ThC.

**Figure 4:**
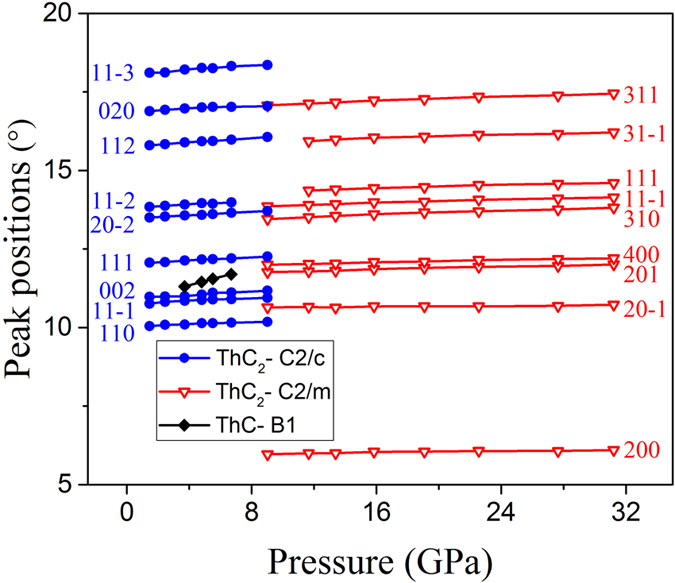
Peak positions of the XRD patterns as a function of pressure.

The micro-focused X-ray diffraction experiment is carried out under pressure up to 50 GPa. The XRD patterns are shown in Fig. 3, and the corresponding peak positions are also plotted as a function of pressure in Fig. 4. At the beginning, there is preset pressure of 1.45 GPa in the DAC. The diffraction pattern of the ThC_2_ sample matches the ground state C2/c phase. As pressure goes up, the peaks systematically shift to larger angles as the sample is continually compressed. The diffraction pattern remains to be C2/c phase up to 6.70 GPa. As the pressure reaches 9.02 GPa, most of C2/c peaks still exist while peaks from C2/m phase appear with weaker intensities. When the pressure reaches 11.66 GPa, only peaks from C2/m phase can be found, and their intensities become stronger. Hence we can conclude that the phase transition of ThC_2_ occurs at ~9.02 GPa from C2/c to C2/m. The coexistence of signals from two phases at this transition point might be attributed to the pressure gradient inside the sample chamber, possibly resulting from the imperfect hydrostaticity of silicon oil and the structural defects of the sample itself.

There is also a small peak at ~11.5 degree on the diffraction profiles between 3.71 and 6.70 GPa. It doesn’t belong to the ambient phase C2/c, and its peak position is close to peak (201) of the new high pressure phase C2/m. However there are no other C2/m signals at this pressure range. What’s more, the intensity of this peak seems to be stable as pressure increases. Hence, it is unlikely that this diffraction peak comes from the C2/m phase. We also check the diffraction signal of the gasket (stainless steel) and rule out this possibility. Based on the sample synthesis process and our study on the other material ThC^18^, we conjecture that this peak comes from (111) of ThC-B1 phase, the strongest peak of ThC-B1 phase at this pressure range. Another evidence of the origin of this peak comes from Fig. 4, where this peak position shifts to larger angle under increasing pressure much more rapidly compared to the two phases of ThC_2_, indicating a different material. We do not see this signal at higher pressures, because peak (201) of ThC_2_ - C2/m phase dominates this position. And the absence of it at lower pressure points might result from the uncontrollable sampling volume during micro-focused XRD measurement at each pressure point and the tiny amount of ThC is small.

**Figure 5:**
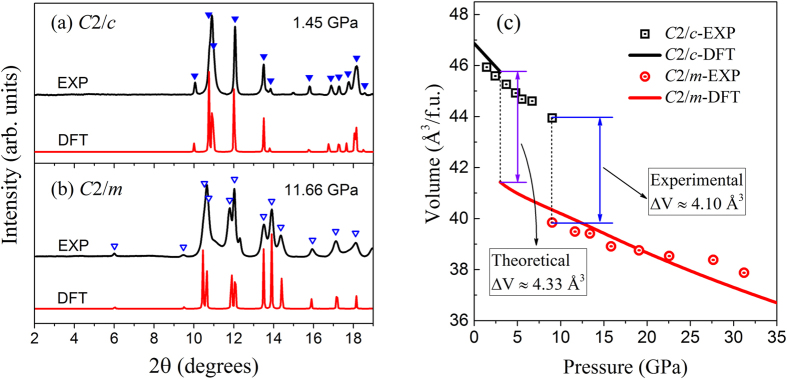
XRD patterns and volume versus pressure relation. Integrated X-ray diffraction patterns (black lines) obtained with subtraction of the background for (**a**) C2/c and (**b**) C2/m phases of ThC_2_ at selected pressure. Simulated powder diffraction patterns (red lines) using atomic positions derived from DFT-optimized structures are shown for comparison. Solid and hollow triangles indicate peaks that belong to the C2/c and the C2/m phases, respectively. (**c**) Experimental measured and DFT calculated volume versus pressure relation for the C2/c and C2/m phases. ∆V is the volume collapse at the transition pressure.

We compare the diffraction pattern obtained from the experiment with the simulated one for the C2/m phase as well as the ground phase in Fig. 5(a) and (b). The parameters of the crystal structure are obtained through the Rietveld refinement and compared with calculations in Table 1 together with previous work^13^,^14^,^15^,^21^. The experimental results agree very well with our theoretical predications, with discrepancy within 0.3%. The volume per f.u. in two states are also calculated based on the experimental data and the theoretical simulation, as seen in Fig. 5(c). The abrupt drop of this value at transition point indicates the occurring of a first order transition. The volume collapse through the transition is estimated to be around 10%, more specifically 4.10 Å^3^ by experiment and 4.33 Å^3^ by simulation. More structural parameters of the high pressure phases of ThC_2_ at the corresponding pressures are listed in Table 1 and the atomic Wychoff positions of these phases are listed in Table 2.

### Polymerization behaviors of carbon atoms

As carbon has rich chemical bonding features, including *sp*-, *sp*^2^-, and *sp*^3^-hybridized bonds, it can exist as isolated dimer, one-dimensional (1D) chain, two-dimensional (2D) plane, and three-dimensional (3D) network in carbon solids or carbon-based compounds. Especially, carbon-based compounds exhibit unexpected structures and electronic behaviors at high pressure, such as C-C polymerization and superconductivity behaviors^22^,^23^,^24^,^25^.

**Figure 6:**
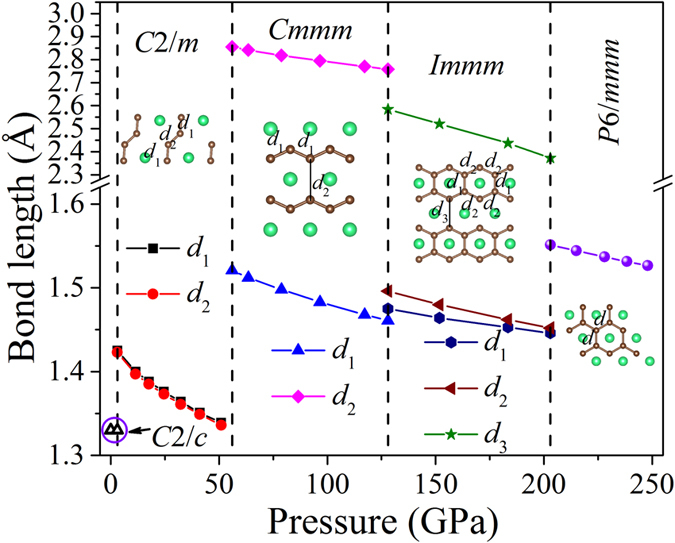
Change of C-C bond lengths in C2/m, Cmmm, Immm and P6/mmm with pressure increasing. The green and brown balls represent Th and C atoms, respectively.

**Figure 7:**
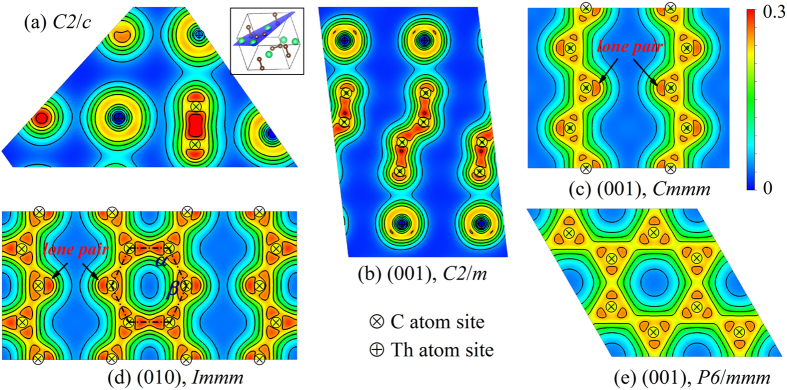
Charge density distribution of various phases ThC_2_. (**a**) The plane as shown in the inset of crystal structure of C2/c at zero pressure, (**b**) the (001) plane of C2/m at 3 GPa, (**c**) the (001) plane of Cmmm at 56 GPa, (**d**) the (010) plane of Immm at 128 GPa and (**e**) the (001) plane of P6/mmm at 203 GPa. The contour interval is 0.05 electrons/Bohr^3^.

For understanding the drive of the phase transitions, the evolution of the carbon-carbon bond under increasing pressure is the key. From low- to high-pressure phase, the C-C bond lengths as a function of pressure are illustrated in Fig. 6, and the charge density distributions of various phases are shown in Fig. 7. One can see that the bonding behavior changes dramatically due to the external pressure.

As in Fig. 6, the bond length of discrete C-C dumbbell is 1.33 Å in C2/c phase at ambient conditions. With pressure increasing, the distance between discrete dumbbells gradually decreases, while the bond length within the dumbbell, i.e. the C-C bond, abruptly increases at the transition pressure from C2/c to C2/m. The elongation and thus weakening of the C-C bond results in the formation of carbon atomic chain-like tetramer in C2/m phase. As pressure goes further up, carbon atomic chain-like tetramers tend to organize in order, and the 1D zigzag carbon chain forms, transforming C2/m into Cmmm. Then, both C-C bond length *d*_1_ and distance between carbon chains *d*_2_ in Cmmm continue decreasing as pressure increases. This leads to the formation of the graphene-like ribbon with six-membered carbon ring in Immm, as shown in Fig. 6. Under further compression, the difference of C-C bond lengths gradually decreases in the Immm phase, and then the crystal transforms to P6/mmm phase, in which the regular hexagonal carbon ring forms. To sum up, under external compression, dumbbell-type carbon in ThC_2_ is polymerized first into ordered chain-like tetramer and further into 1D zigzag chain and then into graphene-like ribbon and eventually into 2D graphite sheet. The similar pressure-induced carbon polymerization phenomenon is also observed in alkaline-earth metal and transition metal dicarbides, such as BeC_2_^26^, MgC_2_^25^,^26^, CaC_2_^22^,^23^, SrC_2_^27^, BaC_2_^28^, LaC_2_^29^, etc. Interestingly, the compounds of the rare-earth metal carbide R_2_C_3_ systems (R = Y, La, Sc, Lu, Nd, Eu, and Gd) also have a similar C-C dimer structure under ambient pressure and have the carbon polymerization phenomenon under high pressure^30^. However, the underlying mechanisms behind the polymerization process of the two types of compounds may be different as stoichiometric ratio and bonding character between these two systems are different. Therefore, the C-C dimer polymerizes directly to bare C6 rings in binary sesquicarbides R_2_C_3_ at high pressure^30^, while the bare C6 rings are not seen in the high-pressure phases of ThC_2_.

It is well known that the near spherical distribution of electron density indicates an ionic character, and the electron density along the bond indicates a covalent character^31^. Figure 7(a) demonstrates that the charge density is centered around Th atoms and there is a small amount of charge density between Th and C atoms, while a large amount of density is distributed along the C-C bond in carbon dumbbell. It indicates that the bond between the Th and C has more ionic character, while the C-C bond in carbon dumbbell has strong covalent character. The bond characters can be applied on the chain-like tetramer in C2/m phase as well, with C-C bond angle as 136.5° at 3 GPa. The charge density distribution of the Cmmm shows that C atoms bind together to form 1D zigzag chain with a stable and localized lone-pair (non-bonding state) at the corner, as shown in Fig. 7(c). The angle between the C-C bonds is 130.9° in Cmmm phase at 56 GPa. The C-C bond in the chain shows dominant covalent with weak ionic character, while neither is as strong as the bond in C2/c and C2/m. For the Immm phase, similar to the *γ*-phase of MgC_2_^25^, C atoms form carbon ribbons assembled by six-member carbon rings in which each of the four inner C atoms forms three C-C covalent bonds, while each of the two outer C atoms has one lone pair of electrons and two C-C covalent bonds, as shown in Fig. 7(d). The bonds of the six-member carbon ring form two different angles *α* and *β*, where *α* = 115.9°, and *β* = 128.2°. In the P6/mmm phase, the six-member carbon ring is a regular hexagon, which has six identical C-C covalent bonds, as shown in Fig. 7(e). From the point view of electron density, it can be seen that the covalent character of C-C bond is weakened with the carbon atoms polymerizing together, meanwhile, the C-C bonding become more uniform.

### Dynamical stabilities and vibrational properties

**Figure 8:**
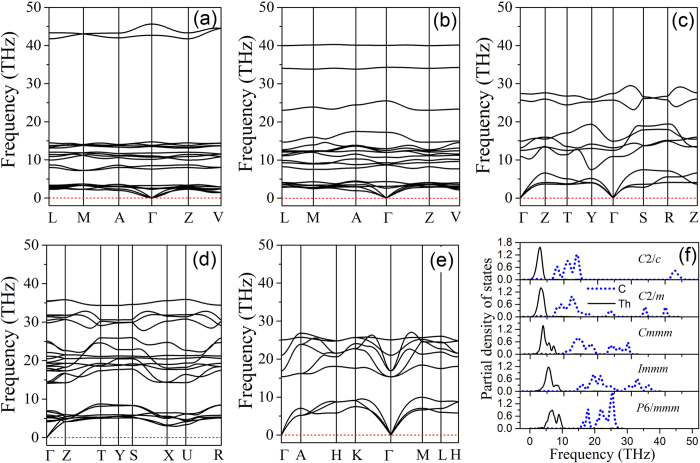
Phonon dispersions density of state. (**a**) C2/c phase, (**b**) C2/m phase, (**c**) Cmmm phase, (**d**) Immm phase and (**e**) P6/mmm phase at 0 GPa, 5 GPa, 56 GPa, 128 GPa and 203 GPa, respectively; And (**f**) Phonon density of state (PDOS) of these phases.

The phonons of a crystal are one of the fundamental subjects when considering the phase stability, phase transitions, and dynamical stability of a crystalline material. The dynamical instability of a crystal is associated with soft phonon modes with imaginary frequency^32^. To check the dynamical stability for the phases of ThC_2_, the phonon dispersion curves along several high-symmetry points of Brillouin zone are calculated, as shown in Fig. 8(a–e). According to our calculations, it is found that all the frequencies for the C2/c, C2/m, Cmmm, Immm and P6/mmm are positive, indicating that these phases are dynamically stable.

Interestingly, there are three distinct groups of phonon branches for the C2/c, separated from each other by large frequency gaps, as illustrated in Fig. 8(a). The highest optical branch lies at least 25 THz above the rest of the optical branches. The phonon DOS splits into three frequency regimes: 0–5 THz, 7–15 THz, and 42–45 THz, as shown in Fig. 8(f). The Th-related vibrations make the dominated contribution to acoustic phonon branches in the low frequency region (LFR) from 0 to 5 THz, and C atoms also make small contribution to this region. The intermediate frequency region (IFR) between 7 and 15 THz is mainly contributed by the vibrations of C atoms due to the smaller weight of C atom. The high frequency region (HFR) between 42 and 45 THz is dominated by the vibrations of C atoms. The phonon branches in HFR are contributed almost completely by vibrations of C-C dumbbell, indicating that there is a strong C-C interaction in C-C dumbbell in C2/c. It is interesting that the frequency of this branch is higher than the corresponding frequency in diamond, graphite, or graphene, which is consistent with the observation that our computed C-C bond length in C2/c (1.33 Å) is noticeably shorter than the nearest neighbour bond lengths in diamond (1.54 Å)^33^, graphite (1.41 Å)^33^ or graphene (1.42 Å)^34^. These results indicate that the C-C pairs of dumbbell in the C2/c phase is more strongly bonded with respect to that in diamond, graphite and graphene.

For the C2/m, the phonon branches in LFR and IFR are similar to that of C2/c. But in HFR, the phonon branches of C2/m split into two parts: one part locates at about 34 THz, the other part at about 40 THz. Both of the two branches are contributed by the vibrations of C atoms, particularly, by the chain-like carbon tetramer in this phase. From Fig. 8(f), it can be noticed that the vibrational modes in LFR and IFR continue shifting to higher frequency as the material transforms from one phase to the other, as a result of increasing external pressure. On the contrary, the vibrational modes in HFR shift to lower frequency in C2/m phase compared to that of C2/c, and disappear in phases of Cmmm, Immm and P6/mmm. The main reason for this is because the pressure-induced carbon polymerization weakens the C-C bonds until they disappear eventually.

## Discussion

Four high-pressure phases of ThC_2_ and their transition sequence are identified as C2/c → C2/m → Cmmm → Immm → P6/mmm as pressure goes up by using *ab initio* total energy calculations in combination with unbiased structure searching method. The critical transition pressures are 3 GPa, 56 GPa, 128 GPa and 203 GPa, respectively. Among these phases, the C2/m is successfully observed for the first time via *in situ* synchrotron X-ray diffraction experiment, and it displays an excellent structural correspondence to our theoretical predictions. The pressure-induced volume collapses are about 9.5%, 11.6%, 2.3% and 2.4% respectively during the transitions with increasing pressure, indicating the first order phase transition.

In the ground-state monoclinic C2/c phase, the carbon atoms exist as isolated C-C dumbbell. With pressure increasing, the dumbbell-type carbon in C2/c is polymerized first into ordered chain-like tetramer in C2/m and further into 1D zigzag chain in Cmmm and then into graphene-like ribbon in Immm and eventually into 2D graphite sheet in P6/mmm. The charge density distribution analysis indicates that the C-C bonds show a dominant covalent character, and it becomes weak as the carbon atoms polymerizing, meanwhile, the strength of different C-C bond tend to becomes more identical.

The phonon dispersions illustrate that all of the phases are thermodynamically stable. The ultra-high frequency vibrations of the C-C dumbbell in the C2/c phase indicate that the C-C bond of the dumbbell is more strongly bonded with respect to that in diamond, graphite, and graphene. From the low-pressure phase to the high-pressure phase, the disappearing of the ultra-high frequency vibration illustrates that the pressure-induced carbon polymerization leads to the gradual weakening and eventual disappearing of the isolated strong C-C bonds. Such insights are crucial to evaluating the behavior and determining the role of ThC_2_ in their applications for advanced nuclear fuels of Generation IV reactor. Although the structural behaviors of this compound under high pressure are extensively studied in this work, some novel properties, e.g. the possible superconductivity under high pressure, as predicted in CaC_2_^22^, have not been clearly understood, which will call for further theoretical and experimental verification and exploration.

## Methods

### Material preparation

Thorium dicarbide was prepared using thorium dioxide (99.99%) and natural graphite powder (99%) as starting materials by carbon thermal reduction method (SDCTM). The ThO_2_ and graphite powder were weighted with C/Th molar ratio of 4.1 for ThC_2_. The powder was mixed and ball-milled for 2 h in ethanol. After ball milling, the slurry was dried at 100 °C for 48 h in a vacuum drier. Then the dried mixture was pressed into green pellets with 5 mm in diameter and 10 mm in height. Finally, the green pellets were sintered at 1950 °C with the vacuum of 1.3 × 10^−3^ Pa for 30 min. After cooling down, the sintered specimens were immersed in cyclohexane to prevent oxidizing and hydrolyzing.

### Experiments

In order to obtain fine powder for X-ray diffraction (XRD) characterization, the bulk sample was ground into sub-micro sized particles (200–300 nm) within silicon oil to avoid deliquescing. A Mao-Bell type symmetric diamond-anvil cell with a pair of 300 μm culet sized diamond anvils was used to generate high pressure environment for thorium carbide. A sample chamber was created by drilling a hole in diameter of 150 μm into a stainless steel gasket pre-indented in thickness of 45 μm. The powder mixed with silicon oil was loaded in the center of sample chamber with the silicon oil as pressure transmitting medium. Three ruby spheres (8–10 μm in diameter) as the pressure standards were placed in the chamber dispersed. *In situ* high-pressure XRD measurements were carried out at BL15U station at Shanghai Synchrotron Radiation Facility (SSRF). The monochromatic x-ray beam at wavelength of 0.6199 Å was focused in size of ~3 μm (vertical) × 2.5 μm (horizontal) in full width at half maximum (FWHM). The diffraction patterns were collected using a MAR345 image plate, with typical exposure time 20 to 60 seconds. Each diffraction pattern was collected after the pressure was adjusted and stabilized to ensure steady pressure during XRD measurements. The two-dimensional diffraction patterns were integrated with the FIT2D program^35^ to produce diffraction curves of intensity versus 2θ.

### Computational details

All of the density-functional-theory (DFT) calculations in this study were carried out using the Vienna *Ab initio* Simulation Package (VASP)^36^,^37^ with the projector augmented wave scheme (PAW)^38^,^39^. The exchange-correlation functional with generalized gradient approximation (GGA) of Perdew-Burke-Ernzerhof (PBE)^40^ was used to solve the Kohn-Sham equations with a kinetic energy cutoff of 600 eV. Since none of the localized 5f-like band is occupied in Th, the DFT with the GGA approximation was proved to be effective enough to describe the structural and vibrational properties of Th-based compounds^11^,^41^,^42^,^43^. The free energy global minimization of ThC_2_ were performed by merging *ab initio* total energy calculations and particle-swarm optimization algorithm^19^ on structural predictions, as implemented in the CALYPSO code^20^. It has been proven to be effective and accurate in predicting the crystal structures of a large variety of materials^44^,^45^,^46^,^47^,^48^,^49^,^50^,^51^,^52^. The structural relaxations were performed allowing the variations of the ionic position, cell volume, and cell shape. The Brillouin-Zone (BZ) of C2/c, C2/m, Cmmm, Immm and P6/mmm phases were sampled respectively with a 8 × 12 × 8, a 4 × 12 × 12, a 12 × 12 × 14, a 16 × 6 × 8, and a 16 × 16 × 12 k-point mesh generated via the Monkhorst-pack scheme^53^. The convergence conditions were set to be 0.001 eV/Å force on each ion and 10^−7 ^eV in the electronic self-consistent loop for the total energy calculations.

The calculations of phonon curves in the BZ and the corresponding phonon density of states (DOS) were carried out by using a supercell approach^54^ as implemented in the PHONOPY code^55^. The 2 × 3 × 2, 1 × 3 × 3, 2 × 4 × 4, 4 × 2 × 2 and 4 × 4 × 3 supercells were created from the optimized crystallographic cell for C2/c, C2/m, Cmmm, Immm and P6/mmm, respectively, in order to ensure the edge lengths of the supercell to be no less than 10 Å. The BZ integration was performed with a k-point mesh of 2 × 2 × 2. The symmetry non-equivalent Th and C atoms were displaced from their equilibrium positions by the amplitude of 0.02 Å to construct the system dynamical matrix D(k). The forces induced by small displacements are calculated within VASP code.

## Additional Information

**How to cite this article**: Guo, Y. *et al*. Pressure-induced structural transformations and polymerization in ThC_2_. *Sci. Rep.* **7**, 45872; doi: 10.1038/srep45872 (2017).

**Publisher's note:** Springer Nature remains neutral with regard to jurisdictional claims in published maps and institutional affiliations.

## Figures and Tables

**Table 1:** Calculated lattice parameters of C2/c, C2/m, Cmmm, Immm and P6/mmm phases of ThC_2_ in comparison with available experimental and calculated data.

	Pressure	*a*_0_ (Å)	*b*_0_ (Å)	*c*_0_ (Å)	*α* (°)	*β* (°)	*γ* (°)
C2/c	0 GPa	6.708	4.267	6.745	90.00	103.81	90.00
		6.691^a^	4.231^a^	6.744^a^	90.00^a^	103.83^a^	90.00^a^
		6.692^b^	4.223^b^	6.744^b^	90.00^b^	103.12^b^	90.00^b^
		6.684^c^	4.220^c^	6.735^c^	90.00^c^	103.91^c^	90.00^c^
		6.6283^d^	4.2069^d^	6.6726^d^			
	1.45 GPa	6.682	4.252	6.718	90.00	103.93	90.00
	Exp.^e^	6.671	4.222	6.722	90.00	103.91	90.00
C2/m	3 GPa	12.059	3.623	3.821	90.00	96.89	90.00
	11.66 GPa	11.877	3.556	3.765	90.00	97.00	90.00
	Exp.^f^	11.867	3.546	3.775	90.00	96.08	90.00
Cmmm	56 GPa	6.974	2.766	3.167	90.00	90.00	90.00
Immm	128 GPa	2.692	7.173	5.367	90.00	90.00	90.00
P6/mmm	203 GPa	2.687	2.687	3.650	90.00	90.00	120.00

^a^Exp., ref. 21;

^b^Exp., ref. 14;

^c^Exp., ref. 15;

^d^Cal., ref. 13;

^e^Our experimental measured results of C2/c phase at 1.45 GPa.

^f^Our experimental measured results of C2/m phase at 11.66 GPa.

**Table 2:** The atomic Wychoff positions of C2/m, Cmmm, Immm and P6/mmm phases.

C2/m	Cmmm	Immm	P6/mmm
Th: 4i (0.86369, 0.5, 0.27907)	Th: 4 g (0.0, 0.0, 0.5)	Th: 4 g (0.0, 0.77630, 0.0)	Th: 1b (0.0, 0.0, 0.5)
C1: 4i (0.04025, 0.0, 0.15241)	C: 2d (0.20467, 0.5, 0.0)	C1: 4i (0.5, 0.5, 0.86255)	C: 2c (1/3, 2/3, 0.0)
C2: 4i (0.15888, 0.0, 0.22237)		C2: 4j (0.5, 0.0, 0.75928)	
